# Radiation Pneumonitis in Thoracic Cancer Patients: Multi-Center Voxel-Based Analysis

**DOI:** 10.3390/cancers13143553

**Published:** 2021-07-15

**Authors:** Giuseppe Palma, Serena Monti, Roberto Pacelli, Zhongxing Liao, Joseph O. Deasy, Radhe Mohan, Laura Cella

**Affiliations:** 1 Institute of Biostructures and Bioimaging, National Research Council, 80145 Napoli, Italy; serena.monti@ibb.cnr.it; 2 Department of Advanced Biomedical Sciences, University of Naples “Federico II”, 80131 Napoli, Italy; roberto.pacelli@unina.it; 3 Department of Radiation Oncology, MD Anderson Cancer Center, Houston, TX 77030, USA; zliao@mdanderson.org; 4 Department of Medical Physics, Memorial Sloan Kettering Cancer Center, New York, NY 10065, USA; DeasyJ@mskcc.org; 5 Department of Radiation Physics, MD Anderson Cancer Center, Houston, TX 77030, USA; rmohan@mdanderson.org

**Keywords:** radiation pneumonitis, thoracic cancer, voxel-based analysis, probabilistic independent component analysis, connectograms

## Abstract

**Simple Summary:**

The pathophysiology of radiation pneumonitis (RP) after thoracic cancer radiation treatments is still not completely understood although the identification of underlying RP mechanisms may improve the therapeutic window of thoracic cancer patients. The aim of our retrospective study was to explore the dose–response patterns associated with RP by a multi-center voxel-based analysis. In a heterogeneously treated population of 382 thoracic cancer patients, we confirmed the previously described heart–lung interaction in the development of RP. The empowerment of VBA with a novel description of dose map spatial properties based on probabilistic independent component analysis (PICA) and connectograms provided valuable additional and independent information on the radiobiology of RP.

**Abstract:**

This study investigates the dose–response patterns associated with radiation pneumonitis (RP) in patients treated for thoracic malignancies with different radiation modalities. To this end, voxel-based analysis (VBA) empowered by a novel strategy for the characterization of spatial properties of dose maps was applied. Data from 382 lung cancer and mediastinal lymphoma patients from three institutions treated with different radiation therapy (RT) techniques were analyzed. Each planning CT and biologically effective dose map (α/β = 3 Gy) was spatially normalized on a common anatomical reference. The VBA of local dose differences between patients with and without RP was performed and the clusters of voxels with dose differences that significantly correlated with RP at a *p*-level of 0.05 were generated accordingly. The robustness of VBA inference was evaluated by a novel characterization for spatial properties of dose maps based on probabilistic independent component analysis (PICA) and connectograms. This lays robust foundations to the obtained findings that the lower parts of the lungs and the heart play a prominent role in the development of RP. Connectograms showed that the dataset can support a radiobiological differentiation between the main heart and lung substructures.

## 1. Introduction

Radiation therapy (RT) represents a fundamental treatment strategy in thoracic oncology. Radiation-induced morbidities—including radiation pneumonitis (RP)—may, however, limit the effectiveness of RT [1,2]. The current clinical practice in treatment planning relies on safe parameters such as mean lung dose (MLD) and lung volume receiving at least a certain level of dose (e.g., V_20 Gy_) to estimate lung toxicity risk by assuming that the entire lung tissue shares the same radiobiological patterns [3]. Moreover, several other variables have been shown to be predictive, including the equivalent uniform dose parameter.

Regional difference in lung radiosensitivity is an old story [4,5,6]. Indeed, while a commonly accepted pathophysiological picture considers the lungs as parallel organs, the higher incidence of lung injury in the lower lobes, together with the differences in regional functionality revealed by nuclear imaging studies, indicates highly heterogeneous structural composition, functional capacity, and sensitivity to radiation. A greater risk of RP after irradiation of caudally located lung tumors has been acknowledged in [7]; however, the underlying local mechanisms still elude a thorough understanding.

The momentum of technological changes, including particle beam therapy [8] or hypofractionated RT [9], of the last decade in radiation therapy poses new challenges in outcome modeling and at the same time emphasizes the limit of the traditional dose-volume histogram (DVH)-based toxicity analysis and the normal tissue complication probability (NTCP) modeling philosophy [10,11].

In this study, we investigate the thoracic dose–response patterns for RP in patients treated for thoracic malignancies taking advantage of the pooled data analysis of different thoracic tumors and different RT modalities. A novel approach based on spatial dose distribution analysis—namely, voxel-based analysis (VBA)—is performed to evaluate the significance of dose differences between groups of patients at a voxel level [12,13]. In addition, we apply an innovative characterization of the spatial properties of the dose distribution, which allows to assess the robustness of regional VBA inferences based on probabilistic independent component analysis and dosimetric connectograms [14].

## 2. Materials and Methods

### 2.1. Patients

We retrospectively analyzed 382 patients from 4 different groups of patients receiving thoracic RT with different treatment modalities at different institutions. A group of patients was treated for Hodgkin lymphoma (HL) at the University “Federico II” of Napoli (Comitato Etico per le Attività Biomediche, IRB 222-10) with 3D conformal RT (3D-CRT) [15,16]. A second group was treated for locally advanced non-small cell lung cancer (NSCLC) at the University of Texas MD Anderson Cancer Center of Houston (IRB 2008-0133) with image-guided intensity-modulated RT (IMRT), and a third one with passive-scattering proton therapy (PSPT) [17,18]. Lastly, a group of patients was treated for NSCLC at the Memorial Sloan Kettering Cancer Center of New York (IRB #16-142) with stereotactic body RT (SBRT) [19]. All patients were scored for RP graded according to the National Cancer Institute’s Common Terminology Criteria for Adverse Events (CTCAE) version 3.

#### 2.1.1. 3D Conformal Radiation Therapy

Ninety-eight patients received supradiaphragmatic involved-site RT with a median total dose of 30.6 Gy (range: [20.8, 45.0] Gy) in daily fractions of 1.5 to 1.8 Gy. Treatment plans were designed in XiO (Elekta AB, Stockholm, Sweden) using the multigrid superposition dose calculation algorithm. Dose maps were obtained with a dose grid size of 3.0 mm × 3.0 mm × 5.0 mm.

#### 2.1.2. Intensity-Modulated Radiation Therapy

A group of 114 patients was treated for locally advanced non-small cell lung cancer (NSCLC) at the University of Texas MD Anderson Cancer Center of Houston according to an institutional review board-approved protocol (2008-0133) with image-guided intensity-modulated RT (IMRT). Patients received a prescribed dose of 66 or 74 Gy, given in 33 or 37 conventional daily fractions (2 Gy). Plans were designed in Pinnacle^3^ (Philips Medical Systems, Andover, MA, USA) using the convolution/superposition dose calculation algorithm. Dose maps were obtained with a dose grid size of 2.0 mm × 2.0 mm × 2.5 mm.

#### 2.1.3. Stereotactic Body Radiation Therapy

One hundred and six patients were treated at the Memorial Sloan Kettering Cancer Center of New York with stereotactic body RT (SBRT) for NSCLC. Patients received a median total dose of 50 Gy (range: [40, 54] Gy) in median 4 fractions (range: [3, 5]). Plans were designed in Eclipse v.13 (Varian Medical Systems, Palo Alto, CA, USA) using the AAA dose calculation engine. Dose maps were obtained with a dose grid size of 1.0 mm × 1.0 mm × 2.0 mm.

#### 2.1.4. Passive-Scattering Proton Therapy

A group of 64 patients was treated for locally advanced NSCLC at the University of Texas MD Anderson Cancer Center of Houston according to an institutional review board-approved protocol (2008-0133) with image-guided passive-scattering proton therapy (PSPT). All patients received a prescribed dose of 66 or 74 Gy, given in 33 or 37 conventional daily fractions. Relative biological effectiveness (RBE) was set to 1.1. Plans were obtained by using the proton convolution superposition algorithm implemented in the Eclipse proton therapy planning system (Varian Medical Systems, Inc., Palo Alto, CA, USA). Dose maps were obtained with a dose grid size of 2.0 mm × 2.0 mm × 2.5 mm.

More details on the protocols and treatment characteristics for each cohort are published elsewhere [3,13,18,19].

Physical dose maps were corrected for different fractionation schemes by computing the biologically effective dose (BED) according to an α/β ratio of 3 Gy.

### 2.2. Spatial Normalization

To allow for voxel-wise comparison of the BED distributions given to different patients, the 382 BED maps were spatially normalized on the common anatomical reference provided by the extended cardiac torso (XCAT) synthetic CT phantom [20]. Briefly, for each patient, the planning CT was registered onto the synthetic CT provided with XCAT after tumor masking [21] via the B-spline elastic image registration algorithm implemented in Elastix [22]. Then, the obtained deformation field was applied to the BED map [23].

### 2.3. Characterization of Spatial Properties of Dosimetric Data

The homogeneity of the VBA statistical power in the analyzed anatomical region (i.e., the thorax) strongly depends on the uniformity of voxel-wise dose moments. In principle, homogeneous mean (*μ*) and standard deviation (*σ*) maps rule out the hypothesis that relevant radiosensitive regions were dampened in the VBA results due to the non-uniform variability of the dose maps. Consequently, *μ* and *σ* maps of normalized BED distributions were computed voxel by voxel over the patients and their uniformity was quantitatively evaluated by the Michelson contrast, which for a function I can be computed as:

(1) $$C_{M}=\frac{I_{\max}-I_{\min}}{I_{\max}+I_{\min}}$$

where $I_{\max}$ and $I_{\min}$ are the highest and lowest values of the function. For a positive-valued function I (such as *μ* and *σ* maps), $0\leq C_{M}\leq 1$. For a given fraction 0<f≤1 of the volume V of the support (i.e., the analyzed organ or anatomical apparatus) of I, $C_{M}\left[I\right]\left(f\right)$ can be defined as the minimum $C_{M}$ assumed by the restrictions of I over the subsets of V $\left\{S_{i}\;|\;∥S_{i}∥=f∥V∥\right\}$. Since $C_{M}\left[I\right]\left(f\right)$ is a monotonically increasing function, a summary description of the uniformity of I is provided by its area under curve (AUC). The AUC value of $C_{M}\left[I\right]\left(f\right)$ is 0 for the constant maps and tends to 1 for highly inhomogeneous maps.

Moreover, the spatial resolution of the VBA results (maps of general linear model—GLM—coefficients and their significance) may be affected by the correlation between the doses delivered to different anatomical substructures (i.e., pericardium, heart chambers and walls, and lung segments and lobes). Indeed, small-scale radiobiological patterns—anyway larger than the nominal resolution of the dose grid—could be captured by a VBA provided that the correlation length in the different dose distributions is accordingly short. The spatial independence of the doses delivered to each substructure was investigated by means of probabilistic independent component analysis (PICA) [24] and connectogram analysis [25]. The PICA blindly infers the model order of the analyzed dataset, which corresponds to the number of statistically significant independent spatial maps that generate the input. When applied to a dose distributions dataset (in the form of *n* [patient] × *M* [voxel]), a PICA model order comparable to the cohort size *n* points out a significantly mutual independence between patients’ dose distributions and allows for a valuable VBA inference. The connectograms, instead, highlight the most relevant associations between each pair of substructures according to the pairwise significance of the Spearman correlation between the mean doses related to the substructures: the VBA will have more chance to discriminate the GLM properties arising from each substructure in correspondence of a weaker correlation. In the connectogram rings, outwards from the center, the average and standard deviation across patients of the mean doses within each substructure and the average across patients of the dose standard deviations within each substructure were presented.

### 2.4. Voxel-Based Statistical Analysis

The performed VBA involved the following statistical analysis at a voxel level [26]: a non-parametric permutation test based on the threshold free cluster enhancement of a maximum-*T* statistics for GLM was used to assess the regional dose differences between patients who developed any-grade RP and who did not. The significance map obtained by the VBA was, thus, implicitly corrected for multiple comparisons. In addition, non-dosimetric variables, which correlated with the considered outcome at a backward stepwise multivariable (MVA) logistic regression model [27], were included in the GLM as nuisance variables. Clusters of voxels with significance level *p* < 0.05 (*S*_0.05_) were generated from the VBA significance map and the mean doses in these clusters were computed.

## 3. Results

**Figure 1:**
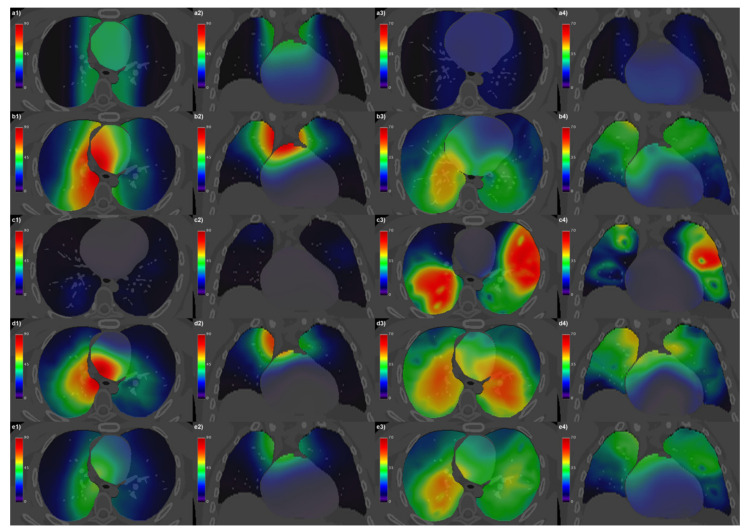
Axial (**columns 1** and **3**) and coronal (**columns 2** and **4**) CT views of the reference anatomy fused with mean (**columns 1** and **2**) and standard deviations (**columns 3** and **4**) of BED maps computed voxel by voxel over the patients for the Hodgkin lymphoma group (**row a**), non-small cell lung cancer (NSCLC) patients treated with intensity-modulated radiation therapy (**row b**), NSCLC patients treated with stereotactic body RT (**row c**), NSCLC patients treated with passive-scattering proton therapy (**row d**), and the whole cohort of patients (**row e**).

**Figure 2:**
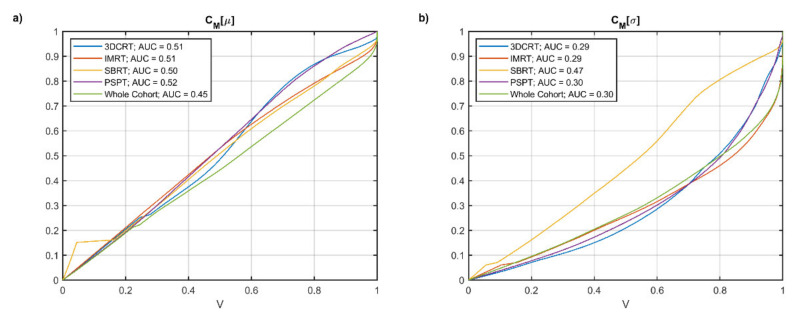
Plots of $C_{M}\left[\mu \right]$ (**a**) and $C_{M}\left[\sigma \right]$ (**b**) as function of the fractional volume V for the different groups of patients and the entire cohort.

The dose *μ* and *σ* maps computed voxel by voxel over the patients for each separate group and for the entire cohort are displayed in Figure 1. The analysis of the uniformity of *μ* and *σ* (Figure 2) shows that the entire cohort overall exhibits consistently lower $C_{M}\left[\mu \right]$ values and a satisfactorily low AUC value of $C_{M}\left[\sigma \right]$ compared with each separate group of patients.

**Figure 3:**
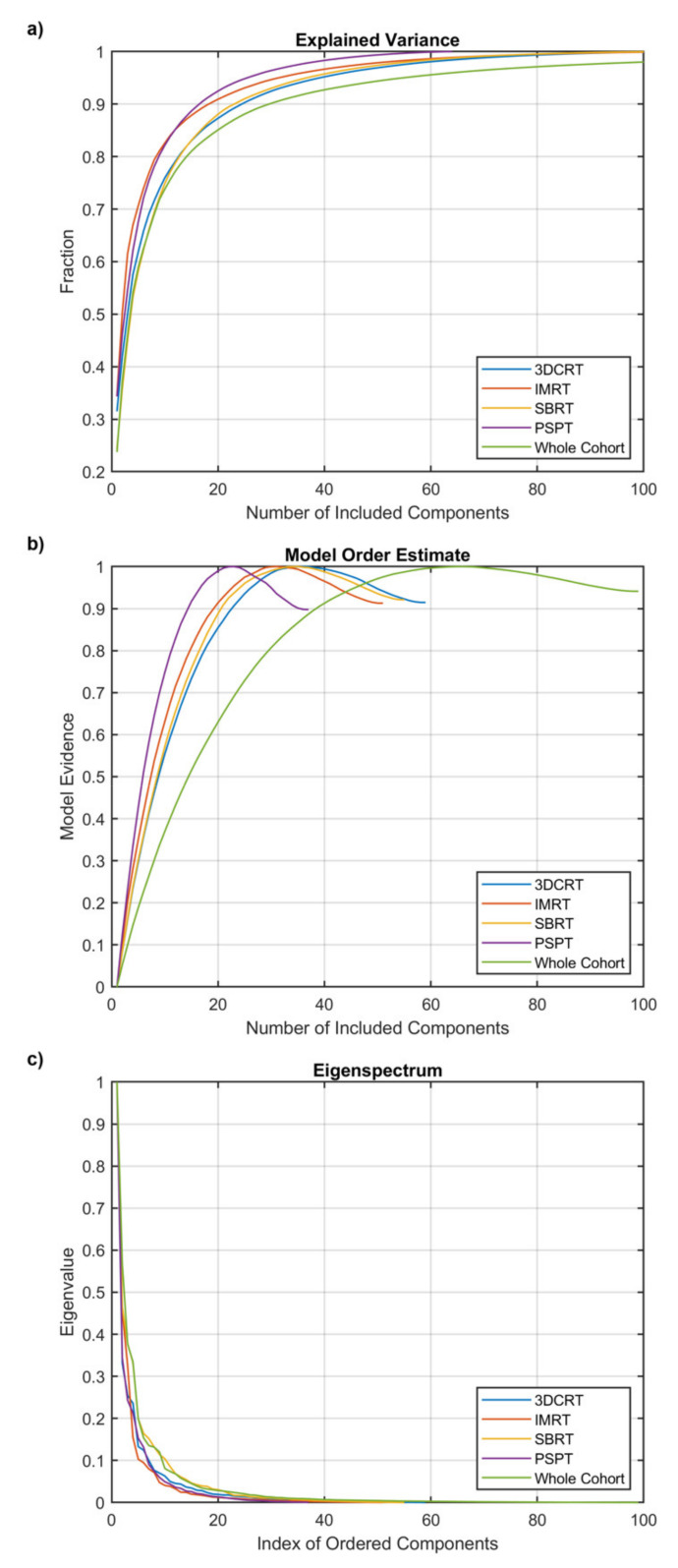
Probabilistic independent component analysis (PICA) order estimation for the analyzed groups of patients. (**a**,**b**) show the explained variance and the model evidence as a function of the included number of components; (**c**) shows the eigenvalue of each component.

**Figure 4:**
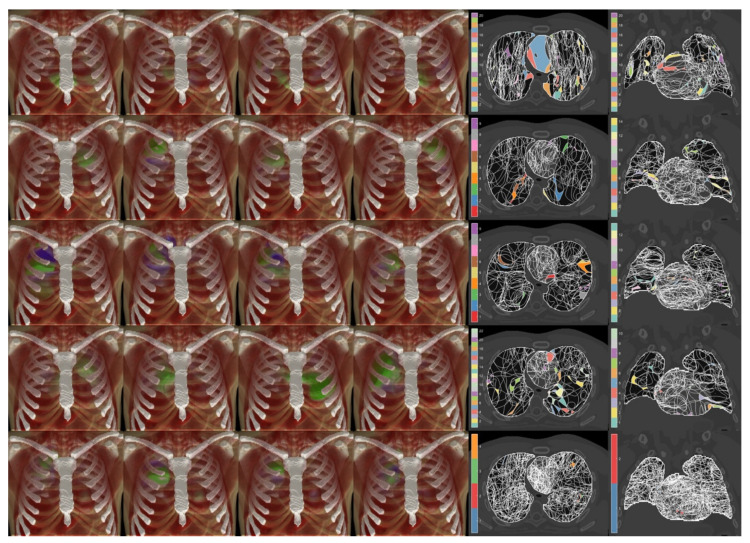
Probabilistic independent component analysis (PICA) of the dose distributions of Hodgkin lymphoma patients (**first row**), non-small cell lung cancer (NSCLC) patients treated with intensity-modulated radiation therapy (**second row**), NSCLC patients treated with stereotactic body RT (**third row**), NSCLC patients treated with passive-scattering proton therapy (**fourth row**), and the whole cohort of patients (**fifth row**). For each row, the first four PICA components are displayed anchored to the underlying patient anatomy, as well as the axial (**fifth column**) and coronal (**sixth column**) views of the PICA component patches exceeding the 95% confidence interval of the normal distribution (sets of patch intersections marked with the same color are connected in the 3D domain).

The PICA detected increasing model orders (Figure 3) for PSPT (23, corresponding to 36% of the number of patients), IMRT (31, 27%), SBRT (35, 33%), and 3D-CRT (36, 37%) groups. The model order for the whole cohort was 66, which corresponds to 17% of the patient number. While we can observe that the PICA components are spread throughout the heart and lungs, they exhibit apparent patterns specific to the irradiation modalities, most clearly highlighted by the patches associated with a unique combination of supra-threshold PICA components (Figure 4). In particular, the 3D-CRT modality for Hodgkin lymphoma patients is visually characterized in the axial view by a strong antero-posterior field direction, with a massive coverage of the mediastinum, which appears indeed to be covered by a few patches layered in the cranio-caudal direction. Notably, the number of patches sensibly increases moving from the single groups to the entire cohort, and the resolution inhomogeneities, specific to the irradiation modalities, are largely washed out throughout the field-of-view in the merged dataset.

**Figure 5:**
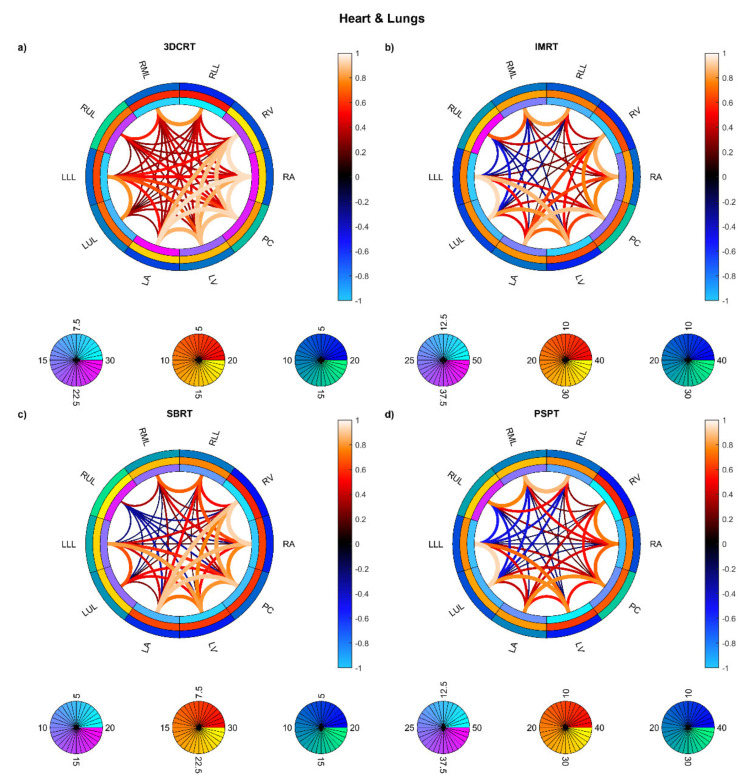
Connectograms in heart and lung substructures for the four groups of patients: Hodgkin lymphoma group—3DCRT (**a**), non-small cell lung cancer patients treated with intensity-modulated radiation therapy—IMRT (**b**), NSCLC patients treated with stereotactic body RT—SBRT (**c**), NSCLC patients treated with passive-scattering proton therapy—PSPT (**d**). The pairwise Spearman correlation coefficients between mean biological effective dose (BED) values to the reported substructures are represented by the lines within the rings. From inside to outside, the rings represent the average of the substructure mean BEDs, standard deviation of the substructure mean BEDs, and average of the dose standard deviations within the substructure. BED is expressed in Gy. Abbreviations: LLL: left lung lobe; LUL: left upper lobe; RLL: right lower lobe; RML: right middle lobe; RUL: right upper lobe; LA: left atrium; LV: left ventricle; RA: right atrium; RV: right ventricle; PC: pericardium.

**Figure 6:**
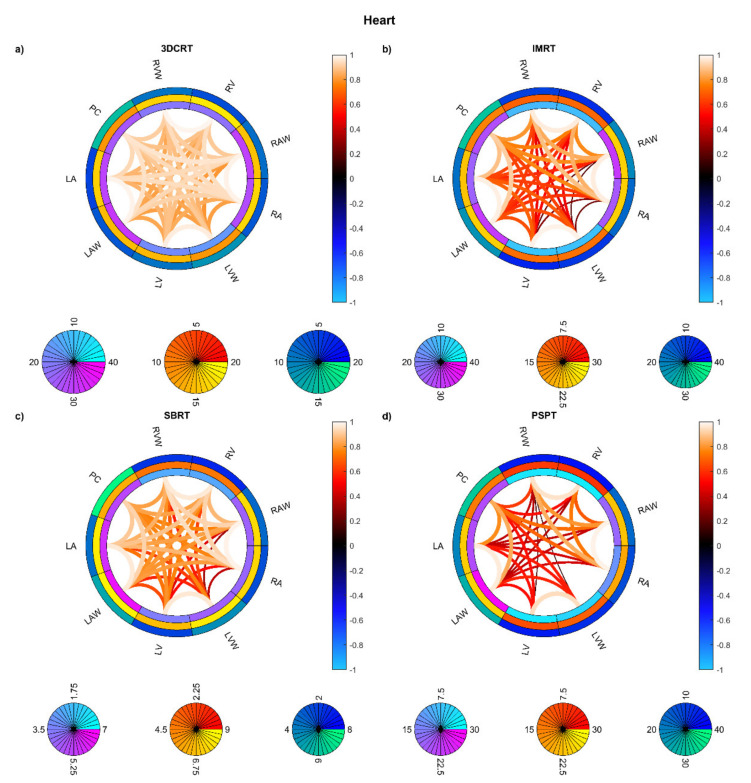
Connectograms in heart substructures for the four groups of patients: Hodgkin lymphoma group—3DCRT (**a**), non-small cell lung cancer patients treated with intensity-modulated radiation therapy—IMRT (**b**), NSCLC patients treated with stereotactic body RT—SBRT (**c**), NSCLC patients treated with passive-scattering proton therapy—PSPT (**d**). The pairwise Spearman correlation coefficients between mean biological effective dose (BED) values to the reported substructures are represented by the lines within the rings. From inside to outside, the rings represent the average of the substructure mean BEDs, standard deviation of the substructure mean BEDs, and average of the dose standard deviations within the substructure. BED is expressed in Gy. Abbreviations: LA: left atrium; LAW: left atrium wall; LV: left ventricle; LVW: left ventricle wall; RA: right atrium; RAW: right atrium wall; RV: right ventricle; RVW: right ventricle wall; PC: pericardium.

**Figure 7:**
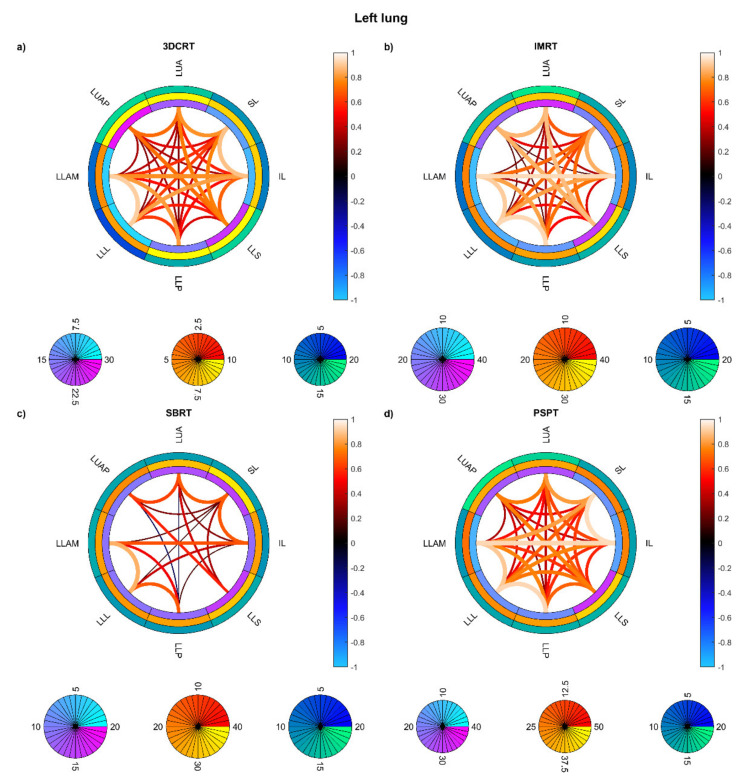
Connectograms in left lung substructures for the four groups of patients: Hodgkin lymphoma group—3DCRT (**a**), non-small cell lung cancer patients treated with intensity-modulated radiation therapy—IMRT (**b**), NSCLC patients treated with stereotactic body RT—SBRT (**c**), NSCLC patients treated with passive-scattering proton therapy—PSPT (**d**). The pairwise Spearman correlation coefficients between the mean biological effective dose (BED) values to the reported substructures are represented by the lines within the rings. From inside to outside, the rings represent the average of the substructure mean BEDs, standard deviation of the substructure mean BEDs, and average of the dose standard deviations within the substructure. BED is expressed in Gy. Abbreviations: LLAM: left lung lower lobe anteromedial; LLL: left lung lower lobe lateral; LLP: left lung lower lobe posterior; LLS: left lung lower lobe superior; IL: inferior lingula; SL: superior lingula; LUA: left lung upper lobe anterior; LUAP: left lung upper lobe apicoposterior.

**Figure 8:**
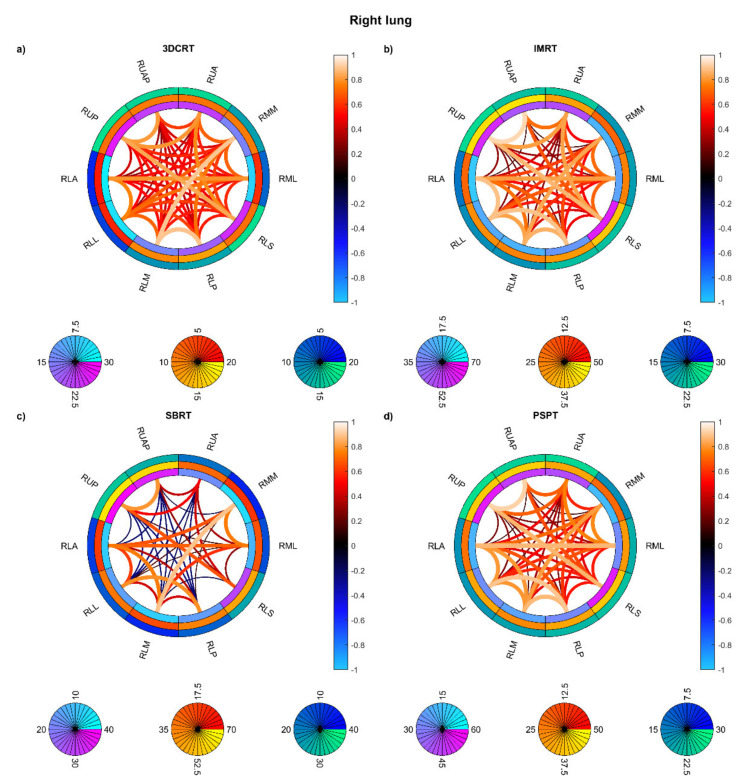
Connectograms in right lung substructures for the four groups of patients: Hodgkin lymphoma group—3DCRT (**a**), non-small cell lung cancer patients treated with intensity-modulated radiation therapy—IMRT (**b**), NSCLC patients treated with stereotactic body RT—SBRT (**c**), NSCLC patients treated with passive-scattering proton therapy—PSPT (**d**). The pairwise Spearman correlation coefficients between the mean biological effective dose (BED) values to the reported substructures are represented by the lines within the rings. From inside to outside, the rings represent the average of the substructure mean BEDs, standard deviation of the substructure mean BEDs, and average of the dose standard deviations within the substructure. BED is expressed in Gy. Abbreviations: RLA: right lung lower lobe anterior; RLL: right lung lower lobe lateral; RLM: right lung lower lobe medial; RLP: right lung lower lobe posterior; RLS: right lung lower lobe superior; RML: right lung middle lobe lateral; RMM: right lung middle lobe medial; RUA: right lung upper lobe anterior; RUAP: right lung upper lobe apical; RUP: right lung upper lobe posterior.

**Figure 9:**
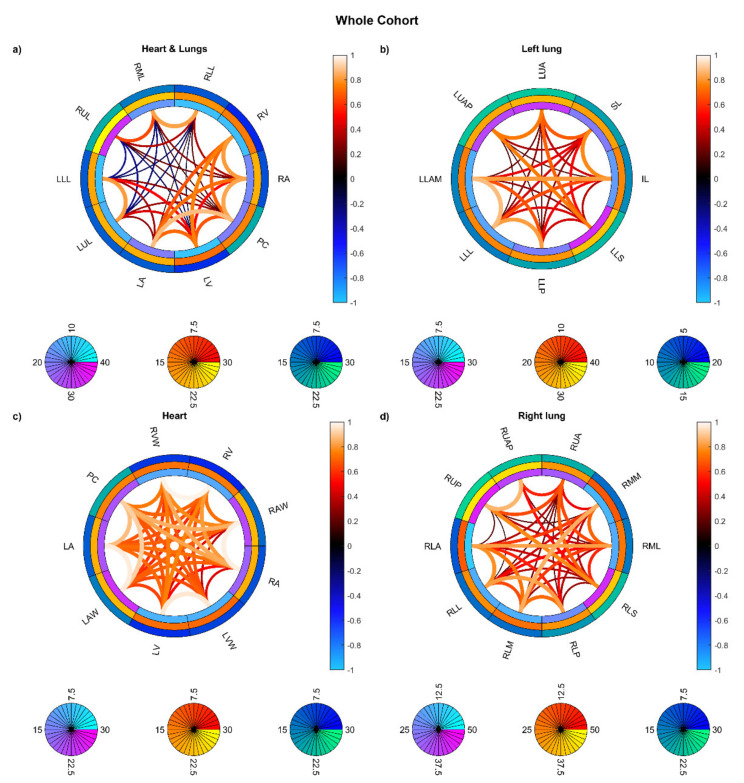
Connectograms for the whole cohort of patients in different sets of substructures: (**a**) heart and lung substructures, (**b**) left lung substructures, (**c**) heart substructures, (**d**) right lung substructures. The pairwise Spearman correlation coefficients between the mean biological effective dose (BED) values to the reported substructures are represented by the lines within the rings. From inside to outside, the rings represent the average of the substructure mean BEDs, standard deviation of the substructure mean BEDs, and average of the dose standard deviations within the substructure. BED is expressed in Gy. Abbreviations: (**a**) LLL: left lung lobe; LUL: left upper lobe; RLL: right lower lobe; RML: right middle lobe; RUL: right upper lobe; LA: left atrium; LV: left ventricle; RA: right atrium; RV: right ventricle; PC: pericardium. (**c**) LA: left atrium; LAW: left atrium wall; LV: left ventricle; LVW: left ventricle wall; RA: right atrium; RAW: right atrium wall; RV: right ventricle; RVW: right ventricle wall; PC: pericardium. (**b**) LLAM: left lung lower lobe anteromedial; LLL: left lung lower lobe lateral; LLP: left lung lower lobe posterior; LLS: left lung lower lobe superior; IL: inferior lingula; SL: superior lingula; LUA: left lung upper lobe anterior; LUAP: left lung upper lobe apicoposterior. (**d**) RLA: right lung lower lobe anterior; RLL: right lung lower lobe lateral; RLM: right lung lower lobe medial; RLP: right lung lower lobe posterior; RLS: right lung lower lobe superior; RML: right lung middle lobe lateral; RMM: right lung middle lobe medial; RUA: right lung upper lobe anterior; RUAP: right lung upper lobe apical; RUP: right lung upper lobe posterior.

On the other hand, the connectograms identified different dosimetric connectivity patterns depending on the RT techniques and the diseases, providing a valuable tool to visually assess the spatial properties of dose distribution. The connectograms for different anatomical districts of the thorax (Figure 5) show that the weakest (possibly negative) spatial correlations are found in PSPT and IMRT groups, with slightly higher correlations in SBRT dose distributions. Conversely, the 3D-CRT group exhibits—as expected—positively correlated dose values throughout the heart and lungs. A similar trend is found in the connectograms dedicated to the smallest cardiac substructures (Figure 6), while the smaller irradiation fields of SBRT plans enable a valuable disentanglement of most small lung segments (Figure 7 and Figure 8). When the groups of dose maps are combined in the analysis of the whole cohort, the connectograms reveal an overall improvement in dose resolution over the considered anatomical substructures (Figure 9).

**Figure 10:**
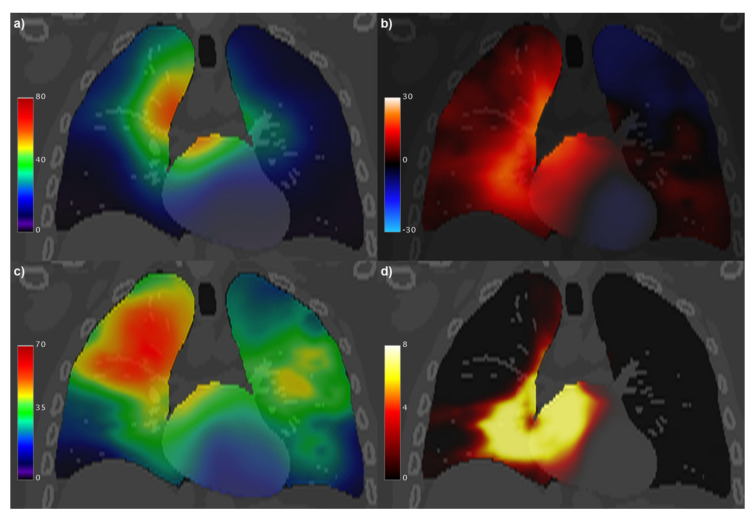
Coronal view of the XCAT computed tomography fused with: (**a**) the voxel-wise mean (*μ*) of the biologically effective dose (BED, in Gy) maps; (**b**) generalized linear model coefficient (in kGy^−1^) associated with BED for the development of radiation pneumonitis; (**c**) the voxel-wise standard deviation (*σ*) of BED maps (in Gy); (**d**) significance of the BED coefficient, expressed as −log *p*.

The incidence of any-grade RP on the entire dataset was 28%. Of the 382 patients, 37 developed grade 1 RP and 50 developed clinically symptomatic RP (i.e., grade ≥ 2). On the whole dataset, age (median: 64 y; range: [13, 93] y) was the only clinical variable significantly correlated with RP (*p* = 0.004) that survived at the MVA analysis and was consequently included in the VBA. VBA highlighted (Figure 10) two largely overlapping *S*_0.05_ clusters (total volume: 551 cc) significantly associated with RP in the lungs (lungs-*S*_0.05_ = 346 cc) and in the heart (heart-*S*_0.05_ = 205 cc). The most represented substructures in the *S*_0.05_ clusters were the right lower and middle lung lobes, the left and right atrium with their walls, and the pericardium. The mean BED in heart-*S*_0.05_ for RP patients was 33.3 Gy, and for patients without RP it was 25.2 Gy. The mean BED in lungs-*S*_0.05_ for RP patients was 38.5 Gy, and for patients without RP it was 25.2 Gy.

## 4. Discussion

Voxel-based analyses were originally introduced in functional imaging to discriminate the local morphological or metabolic patterns between groups of subjects.

In the last decade, techniques developed for imaging VBA have been applied in the field of radiation oncology, and in particular, but not exclusively, to the analysis of radiation-induced toxicity [28,29,30,31,32]. In particular, local dose–response patterns have been evaluated via voxel-wise statistical analysis of dose maps, provided that they were spatially normalized on a common anatomical reference.

VBA applied to both functional imaging and radiobiology typically results in a significance map of radiological or dose differences between groups of patients. In principle, VBA techniques can be formally applied, in a similar way, to both functional imaging and dosimetric datasets; however, there is a substantial difference between the two fields that warrants caution during result interpretation. Indeed, in functional imaging, VBA can identify the metabolic patterns that depend only on the investigated pathophysiology, at least as long as the chosen image contrast, which is usually given by the contrast medium in nuclear medicine and by the pulse sequence in MRI, allows for an effective representation of the underlying functional mechanisms [33,34]. In radiation oncology, on the contrary, VBA can identify those dose patterns that not only depend on the regional radiosensitivity, but are also within the reach of the intrinsic heterogeneity of the dose distributions included in the analyzed dataset.

When applying the VBA to the outcome analysis of radiation treatments, it is therefore essential to account for the intrinsic features of the dose map dataset that could, in principle, limit the very validity of the VBA results. In this study, we tried to preliminarily answer the question, how much radiobiological detail can we identify from a cohort of patients treated with a given radiation therapy technique?

Essentially, we identified two main issues: the power homogeneity and the resolution of VBA, respectively related to the homogeneity of mean and standard deviation of the dose maps and to the correlation between doses to different anatomical structures [14,35]. They seem particularly relevant in the context of thoracic radiation oncology, due to the variety of pathologies and the complexity of pathophysiological pathways proposed in the literature to account for the adverse events in the cardiopulmonary system [15,36,37].

In this context, we exploited for the first time, on a heterogeneously treated population of thoracic cancer patients, a collection of tools devised to assess the spatial properties of dose distributions in order to highlight the power and resolution limits related to the VBAs that can be performed on each dataset [14]. As it has long been expected, it turned out from a rigorously defined analysis that merging different types of dose distributions leads to an improved homogeneity of VBA statistical power as well as to dampened spatial autocorrelation functions. In addition to the straightforward boost of statistical power granted by the increasing number of analyzed patients, which permitted us to obtain by the present study an unparalleled accuracy of the dose–response findings associated with RP of any grade. In particular, it is noteworthy that, despite the valuable independence of the dose values observed in the different substructures in the whole cohort (Figure 9), previously claimed interactions between the heart and the lungs in the RP development [38,39,40,41,42,43] are hereby confirmed. Indeed, we found, in the whole cohort, extended lung and heart regions in which the dose appears significantly correlated with the development of any-grade RP. In these regions an increased mean dose was found in association with RP status: the increase, in patients with RP, was measured to be approximately 13 Gy in lungs-*S*_0.05_ and 8 Gy in heart-*S*_0.05_.

One potential limitation of this work, which may affect both the strictly speaking VBA and our auxiliary analyses of the intrinsic features of the dose map dataset, is the dosimetric uncertainties associated with the use of planning dose maps instead of delivered accumulated dose maps. However, such uncertainties are not group related; consequently no bias is expected in the analysis described here.

## 5. Conclusions

The Michelson contrast of voxel-wise dose moments, as well as the dosimetric PICA and connectograms, represents a valuable toolbox to provide essential insights into the homogeneity and resolution limits inherent to a given dataset of dose maps in the VBA context. Heterogeneous dose patterns, steeper gradients, and smaller hot spots all contribute to a more suitable dataset for dosomic inference. We suggest that a spatial characterization of the dose datasets should constitute an ancillary analysis for every voxel-based analysis in the field of radiation oncology in order to better elucidate the pathophysiological mechanisms underlying radiation-induced morbidity development such as RP.

## Data Availability

The data presented in this study are not publicly available due to restrictions in the Material Transfer Agreements.
